# Malnutrition in Children with Congenital Zika Virus Syndrome: A Systematic Review

**DOI:** 10.1007/s10995-026-04252-5

**Published:** 2026-04-09

**Authors:** Deysiane Santiago da Silva, Letícia Karla Cunha dos Santos, Suamy Sales Barbosa, Ádila Danielly Souza Costa, Nivia Maria Rodrigues Arrais, Karla Danielly da Silva Ribeiro, Márcia Marilia Gomes Dantas Lopes

**Affiliations:** 1 Postgraduate Program in Nutrition, Health Sciences Center, Federal University of Rio Grande do Norte, Natal, Rio Grande do Norte Brazil; 2 Federal University of Rio Grande do Norte, Natal, Rio Grande do Norte Brazil; 3 Department of Nutrition, Health Sciences Center, Federal University of Rio Grande do Norte, Natal, Rio Grande do Norte Brazil; 4 Dr. João Machado General Hospital, Natal, Rio Grande do Norte Brazil; 5 Department of Pediatrics, Health Sciences Center, Federal University of Rio Grande do Norte, Natal, Rio Grande do Norte Brazil; 6 Department of Nutrition, Health Sciences Center, Postgraduate Program in Nutrition, Federal University of Rio Grande do Norte, Natal, Rio Grande do Norte Brazil; 7 Department of Nutrition, Health Sciences Center, Postgraduate Program in Applied Sciences to Women’s Health, Federal University of Rio Grande do Norte, 3000 Senador Salgado Filho Avenue, Natal, Rio Grande do Norte 59078-970 Brazil

**Keywords:** Zika virus infection, Stunting, Underweight, Malnutrition, Infant

## Abstract

**Objective:**

We examined previous studies to describe growth deviations in children with Congenital Zika virus Syndrome (CZS), and to determine the factors associated with malnutrition.

**Study Design:**

Protocol of systematic review registered on the PROSPERO platform under code CRD42023460505. The searches were conducted in SciELO, LILACS, Embase, Scopus, and PubMed/MEDLINE databases, as well as in dissertation and thesis repositories. The eligibility criteria required that the publications must be observational, cross-sectional, cohort, or case-control studies conducted with children with microcephaly associated with CZS, which presented results (z-score) of anthropometric indices of weight-for-age, height-for-age, weight-for-height, and/or body mass index (BMI)-for-age; written in English, Portuguese, or Spanish; and published between 2010 and 2023. The z-score values ​​from anthropometric indices and the factors associated with malnutrition (wasting, underweight, overweight, or stunting) were extracted.

**Results:**

Eleven studies met our inclusion criteria. In these studies, children’s ages ranged from 0 to 48 months. Malnutrition was identified as stunting (14.3% to 57.1%), underweight (14.3% to 54.4%), wasting (4.3% to 48.0%), and, to a lesser extent, as overweight (4.6% to 68.6%). The association of these indices was examined in relation to dysphagia, absence or duration of breastfeeding, delayed introduction of complementary feeding, consumption of ultra-processed foods, and feeding route.

**Conclusion:**

It was possible to identify short stature, wasting, excess weight, and inappropriate eating practices in children with CZS.

## Significance

Children with Congenital Zika Virus Syndrome (CZS) represent a highly vulnerable population. This study synthesizes the available evidence and demonstrates that growth deviations, ranging from stunting and wasting to excess weight, are highly prevalent and markedly heterogeneous across different settings. Importantly, it identifies factors associated with nutritional outcomes, including dysphagia, inadequate breastfeeding practices, delayed or inappropriate introduction of complementary feeding, high consumption of ultra-processed foods, and feeding route. These findings highlight the need for standardized assessments and targeted, multidisciplinary nutritional interventions to improve growth and health outcomes in this group

## Introduction

Brazil was one of the first countries to identify the relationship between Zika virus infection during pregnancy and the birth of newborns with microcephaly. The detection of viral RNA in the amniotic fluid and placenta of pregnant women and brain tissues of neonates and stillbirths highlighted a public health emergency, especially between 2015 and 2019 (Brazil, 2020; Oliveira & Costa, 2016; Salge et al., 2016). Infection during pregnancy may lead to a spectrum of anomalies, which has been called the Congenital Zika virus Syndrome (CZS), with a varied clinical presentation, including microcephaly, which stands out as a risk factor for divergent neurocognitive development of children (Arroyo, 2018; Botelho et al., 2016; Brazil, 2017; Eickmann et al, 2016; Mocelin et al., 2019; Teixeira et al., 2020).

Studies have shown that Zika virus infection during pregnancy can cause several functional alterations, leading to a series of changes in the growth and development of children (Fraga & Varela, 2012; Freitas et al., 2020). Underweight is frequently observed in the first years of life and is associated with the disease itself (Carvalho-Sauer et al., 2020; Oliveira et al., 2020; Santos et al., 2019a; Santos et al., 2021a) however, there is also overweight/obesity (Coelho et al., 2019; Lopes, 2018).

Children with CZS present neurological impairments and frequent clinical complications, such as dysphagia, reflux, epilepsy, constipation, and seizures, that directly compromise feeding and growth. Inadequate feeding practices, including reduced exclusive breastfeeding and early consumption of ultra-processed foods, further increase nutritional vulnerability. Available studies report a high prevalence of stunting and underweight, often worsening over time, highlighting the significant clinical and public health impact of malnutrition in this population. However, evidence remains fragmented, particularly in relation to nutritional diagnosis and monitoring, regarding its magnitude and associated factors. Therefore, a systematic review is needed to synthesize current findings and support evidence-based nutritional care and health policies for children with CZS.

For healthcare professionals, recognizing these patterns is essential for the early identification of nutritional risk, rigorous anthropometric monitoring, prevention of adverse outcomes, and timely implementation of specialized and multidisciplinary care. For mothers and caregivers, access to clear information about nutritional risks and feeding challenges supports informed decision-making, improves feeding practices, and increases engagement in family-centered care focused on skills development. Furthermore, the association between social vulnerability, low social protection, and poorer health outcomes, including lower survival rates among children without financial assistance, highlights the need for integrated approaches that combine medical care, nutritional support, caregiver education, social assistance, and public policies aimed at guaranteeing basic rights, promoting social inclusion, and supporting the healthy growth and long-term development of children with CZS (Arrais et al., 2025; Coelho et al., 2022; Marques et al., 2025; Santos et al., 2019a, 2019b; Tavares et al., 2025). Therefore, this study aimed to examine previous studies to describe growth deviations during childhood in children with microcephaly associated with CZS, and determine the factors associated with malnutrition.

## Materials and Methods

### Search Strategy and Selection Criteria

This study is a systematic review conducted according to the Preferred Reporting Items for Systematic Review and Meta-analysis (PRISMA) (Page et al., 2021), with protocol registered on the PROSPERO platform under code CRD42023460505, with the research questions: what growth deviations are present in children with CZS? and what are the factors related to malnutrition in children with CZS?

The searches were conducted in July 2021 and in January 2023 (update search) in the Scientific Electronic Library Online (SciELO), Latin American Literature in Health Sciences (LILACS), Embase, Scopus, and PubMed/Medline databases, in addition to manual searches in the reference list of the studies included and on the Coordination for the Improvement of Higher Education Personnel (CAPES) repositories for dissertations and theses.

The search strategy used was: (Infant OR Infant, Newborn OR Infant, Large for Gestational Age OR Infant, Low Birth Weight OR Infant, Postmature OR Infant, Premature OR child OR Child, Preschool OR children OR pediatrics OR Neonatology Pediatric OR Emergency Medicine OR Perinatology) AND (Microcephaly OR Microcephalies OR Severe Congenital Microcephaly OR Nervous System Malformations OR Malformations of Cortical Development) AND (Zika virus OR ZikV OR Virus, Zika OR RNA Viruses OR Positive-Strand RNA Viruses) AND (nutritional status OR Diet, Food, and Nutrition OR Health Status OR Nutrition Assessment OR low weight OR Body Weight OR Body Weights and Measures OR Cachexia OR Emaciation OR Leanness OR Underweight OR obesity OR Pediatric Obesity OR Overweight OR Nutritional and Metabolic Diseases) and their respective terms in Portuguese.

The eligibility criteria required that the publications must be observational, cross-sectional, cohort or case-control studies conducted with children with microcephaly associated with CZS, which presented results (z-score) of anthropometric indices of weight-for-age, height-for-age, weight-for-height, and/or body mass index (BMI)-for-age; written in English, Portuguese, or Spanish; and published between 2010 and 2023. Malnutrition was considered when z-score <−2 to weight-for-age (underweight), height-for-age (Low length-for-age or stunting), weight-for-height and/or body mass index (wasting), and/or > + 2 z-score to weight-for-height and/or body mass index (overweight/obesity) (UNICEF et al., 2023).

The records excluded were those in which the studied population did not present microcephaly associated with CZS, experimental works with animals and in vitro models, reviews, opinion articles, and letters to journal editors. Studies were also excluded when the title mentioned other comorbidities or life stages that did not meet the eligibility criteria.

### Data Extraction and Quality Assessment

All records retrieved from the databases were exported, and duplicates were removed. The processes of record identification, screening, and eligibility assessment were conducted independently by DSS and LKCS. Data were extracted from the papers that met the eligibility criteria and were, thus, included in this systematic review. Extracted data containing methodological and outcome variables from each study were as follows: authors, publication year, country, study objective, study design, participants’ age range, total sample size, duration of follow-up, study outcomes (z-score of the anthropometric indices: weight-for-age, height-for-age, weight-for-height and/or BMI-for-age), the proportion of malnutrition (underweight, wasting, stunting, and overweight/obesity), main findings, and the main findings related to malnutrition and its measurements (OR, p value, RR, r^2^). In studies with multiple growth assessments, data extraction was standardized to baseline and end-of-follow-up points.

Children’s nutritional status was classified according to the WHO Child Growth Standards (2006) (World Health Organization, 2006), with adaptations that grouped anthropometric indices with z-scores < − 2 into a single category.

The methodological quality and risk of bias of the cohort and cross-sectional studies were assessed using the Newcastle–Ottawa Scale (Wells et al., 2014) and the Modified Newcastle–Ottawa Scale for Cross-Sectional Studies. Articles with ratings of 0–3, > 3–6, > 6–8, or > 8–9, respectively, were categorized as “poor”, “fair”, “good”, or “excellent” (Supplementary Table 1).

## Results and Discussion

**Fig. 1:**
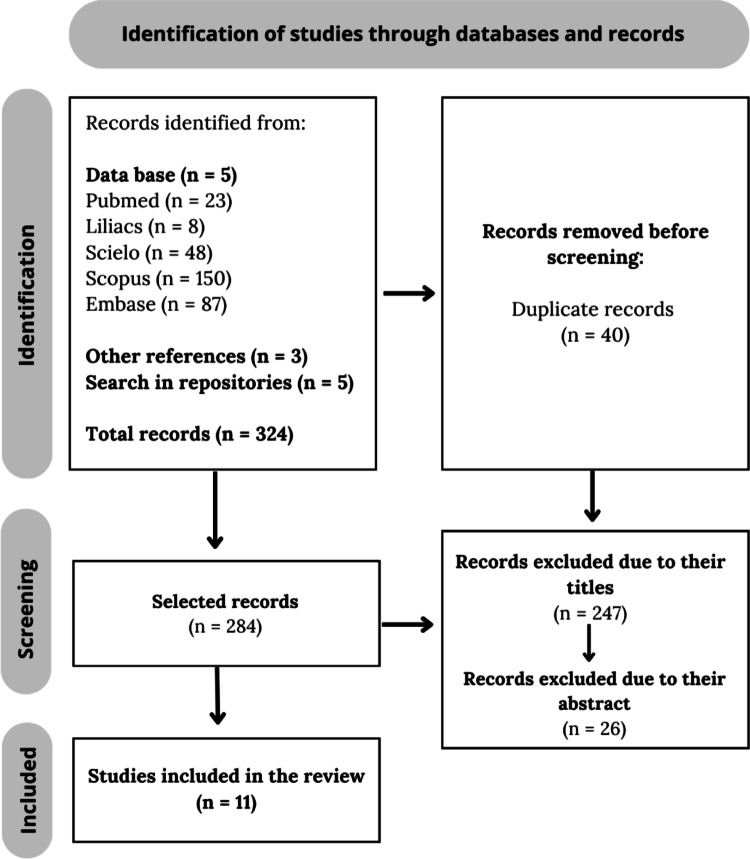
PRISMA flow diagram, used as a selection strategy

The database search identified 324 records. After removal of duplicates, the remaining studies were screened based on titles and abstracts. Following full-text assessment for eligibility, eleven articles were included in the present review (Fig. 1). The extracted data are summarized in Tables 1 and 2.

**Table 1:** Characteristics of the studies included (*n* = 11)

Author (year)/location	Methodological design	Sample size	Gestational age at birth	Objective of the study
Vitorino (2017), Rio Grande do Norte, Brazil	Cross-sectional	Females: 20 Males: 16	N.I.	To analyze the growth and development profile of children with microcephaly associated with CZS.
França et al. (2018), Rio Grande do Norte, Brazil	Cross-sectional	Females and Males: 8	Full-term: 100%	To compare the growth and development of children with CZS to those of typical children.
Lopes (2018), Sergipe, Brazil	Cohort	Females: 49 Males: 35	Preterm: 25,0% Full-term: 75,0%	To evaluate the pattern of growth, development, feeding characteristics, and the occurrence of associated morbidities in children with CZS, from birth to 18 months of life.
Paula (2019), Pernambuco, Brazil	Case-control	Females: 26 Males: 28	Preterm: 10,9% Full-term: 89,1%	To describe the gastric motility, occurrence of diet intolerance, and nutritional status of preschoolers with microcephaly due to CZS.
Coelho et al. (2019), Piauí, Brazil	Cross-sectional	Females: 23 Males: 12	Preterm: 14,2% Full-term: 85,8%	To investigate the nutritional status of newborns with microcephaly due to CZS.
Santos et al. (2019a), Rio de Janeiro, Brazil	Cohort	Females: 33 Males: 32	Preterm: 12,3% Full-term: 87,7%	To describe the nutritional status and feeding practices of children with microcephaly due to CZS at birth and from 12–23 months of age.
Santos et al. (2019b), Rio de Janeiro, Brazil	Cohort	Females: 12 Males: 9	Full-term: 100%	To describe the nutritional profile of newborns with microcephaly and the factors associated with worse outcomes in the first 14 days of life.
Carvalho-Sauer et al. (2020), Bahia, Brazil	Cross-sectional	Females: 21 Males: 25	Preterm: 13,0% Full-term: 87,0%	To evaluate the anthropometric characteristics of children with CZS up to 12 months.
Oliveira et al. (2020), Sergipe, Brazil	Cross-sectional	Females: 24 Males: 21	N.I.	To investigate oral and maxillofacial conditions in children with CZS and the presence of non-nutritive sucking habits, functional habits, and resources related to breastfeeding and nutrition in them.
Aguiar et al. (2022), Rio de Janeiro, Brazil	Cohort	Females: 45 Males: 42	Full-term: 100%	To describe the growth parameters for weight, height and head circumference in children with CZS up to 3 years old.
Tavares et al. (2022), Paraíba, Brazil	Cohort	Females: 50 Males: 67	Preterm: 12,1% Full-term: 87,9%	To describe and analyze changes in anthropometric parameters in children with CZS from birth to 4 years of age.

*N.I* not informed; *CZS* congenital Zika virus syndrome

**Table 2:** Anthropometric data of children with microcephaly associated with CZS in the selected studies (*n* = 11)

Author (year)	Age group	Weight-for-age		Height-for-age		Weight-for-height			BMI-for-age			Main findings/occurrence of deaths	
		Mean z-score	% Malnutrition	Mean z-score	% Malnutrition	Mean z-score		% Malnutrition	Mean z-score		% Malnutrition		
Vitorino (2017)	20.3 months*	N.I	Underweight*	N.I	Stunting*	N.I		Wasting*	N.I		Eutrophy*	Nutritional deficits and changes in developmental milestones were found in children with CZS/No data on deaths.	
França et al. (2018)	20.5 months*	−2.97	Underweight*	N.I.	Stunting*	N.I		Wasting*	N.I		Wasting*	Weight, height, and head circumference were lower in children with CZS (*p* < 0,05)/No data on deaths.	
Lopes (2018)	Follow-up age period: 1–18 months	−1.2 – −1.9^#^	Underweight^#^: 26.5–54.4%	−1.4–2.5^#^	Stunting^#^: 30.9–56.5%	0.69–1.01^#^		Wasting^#^: 4.3–33.0% Overweight/Obesity^#^: 13.2–40.0%	N.I		N.I	Inadequate feeding practices (exclusive breastfeeding, delay in introducing food, and any signs of feeding difficulties)/No data on deaths during the follow-up period.	
Paula (2019)	30 months**	N.I.	Underweight: 38.9%	N.I.	Stunting: 50.0%	N.I.		Wasting: 25.9% Overweight: 10.7%	N.I.		N.I.	ORo: More cases of underweight (*p* = 0.08) and wasting (*p* = 0.02). AR: Higher risk of being overweight (*p* = 0.02). The longer the use of ARs, the higher the weight-for-age and weight-for-height ratios (*p* < 0.001)/No data on deaths.	
Coelho et al. (2019)	Newborns	N.I	Underweight: 40.0%	N.I	Stunting: 51.4%	N.I		Wasting: 31.4% Overweight: 57.2%	N.I		Wasting: 14.3% Overweight/Obesity: 68.6%	Both nutritional deficit (underweight/wasting) and overweight were found in children with CZS/No data on deaths.	
Santos et al. (2019a)	Follow-up age period: 0–23 months	−1.62	Underweight: 20.0–41.5%^#^ Excess weight: 1.5%	−1.94	Stunting: 23.1–56.9%^#^	N.I		Wasting: 29.2% Overweight/Obesity: 4.6%	N.I		Wasting: 24.6% Overweight/Obesity: 6.2%	Inadequate dietary practices (exclusive breastfeeding and intake of UPF), and a positive association between the z-score for height-for-age (OR = 0.606, *p* < 0.0001, weight-for-height (OR = 0.377, *p* = 0.02), weight-for-age (OR = 0.587, *p* < 0.0001), and BMI-for-age (OR = 0.314, *p* = 0.012)/No data on deaths.	
Santos et al. (2019b)	Follow-up age period: First 14 days of life	−2.42	At birth: Underweight: 14.3%	N.I	At birth: Stunting: 14.3%	N.I		At birth: Wasting: 23.8%	N.I		At birth: Wasting: 23.8% Overweight/Obesity: 4.8%	Children with CZS have lower z-scores (*p* = 0.036), and when using infant formula, there was a lower z-score than children on EBF (*p* = 0,035)/No data on deaths during the follow-up period.	
Carvalho-Sauer et al. (2020)	0–12 months	− 0.72	Underweight: 20.4%	− 1.05	Stunting: 39.1%	− 0.43		Wasting: 9.2%	N.I		N.I	There was an inverse association between height-for-age at 12 months and dysphagia (*p* = 0.015), and a direct association between breastfeeding duration and the weight-for-age z score at 3 months (*r* = 0.70; *p* = 0.005) and 6 months (*r* = 0.54; *p* = 0.025)/No data on deaths.	
Oliveira et al. (2020)	0–24 months (17 months**)	N.I.	Underweight**	N.I.	Stunting**	N.I.		Wasting**	N.I.		Thinness**	Children with CZS are more likely to develop dysphagia (OR = 6; 2.53–14.25), a greater chance of not being exclusively breastfed up to 6 months (OR = 1.56; 1.18–2.08), intake of UPF (OR = 1.28;1.01–1.62), and being underweight (OR = 8.33; 2.02–34.45)/No data on deaths	
Aguiar et al. (2022)	Follow-up age period: 0–36 months	−0,93 – −2,15^#^	Underweight (25–36 months): 52.8%	−0,86 – − 2,3^#^	Stunting (25–36 months): 57.1%	0.2 – −1.45^#^		Wasting (25–36 months): 37.1%	N.I.		N.I.	Children with CZS had shorter duration of EBF and BF (*p* < 0.05)/Five deaths (5.7%) occurred during the follow-up period.	
Tavares et al. (2022)	Follow-up age period: 0–48 months	−0.61 – −1.8^#^	Underweight^#^: 10.6% – 46.0%	−0.96 – −2.11^#^	Stunting^#^: 28.6% – 57.0%	N.I.		N.I.	−0.12 – −1.48^#^		Wasting^#^: 26.6% – 48.0%	Children with CZS were associated with shorter birth length (*p* < 0.001), birth weight (*p* < 0.001), and BMI in the second year of life (*p* = 0.04)/Seven deaths (5.9%) occurred during the follow-up period, one in the first year, two in the third year, and four in the fourth year of life.	

^1^Birth weight in grams; ²Length at birth in centimeters; *Mean; **Median; ^#^variation between the follow-up period; CZS – Congenital Zika Virus Syndrome; BMI – Body Mass Index; N.I – Not informed; OR – *Odds Ratio*; ORo – Oral route; AR – Alternative route; UPF – Ultra-processed foods; HC – Head Circumference; BF – Breastfeeding; EBF – Exclusive Breastfeeding. variation between the follow-up period

### General Characteristics and Quality of Studies Included

All included studies were conducted in Brazil and addressed the Zika virus epidemic that occurred between 2015 and 2016. The study populations ranged from newborns to preschool-aged children (0–48 months) and included observational studies of both preterm babies (< 37 weeks of gestation) and full-term babies (≥ 37 weeks of gestation). Small-for-gestational age (SGA) was reported in only three of the included studies. Two studies reported no cases of SGA (0%) (Coelho et al., 2019; França et al., 2018), whereas one study reported a prevalence of 39.1% of participants classified as SGA (Aguiar et al., 2022).

### Anthropometric Nutritional Status of Children with Microcephaly Associated with CZS

The included studies employed multiple anthropometric indices to assess malnutrition, with stunting and underweight being the most frequently identified outcomes, followed by wasting in all studies (*n* = 11). The proportion of nutritional deviations among children ranged from 4% (minimum, wasting) to 57% (maximum, stunting), with stunting consistently presenting higher prevalence rates (exceeding 30.9% of cases) compared with other forms of malnutrition (Table 2). In cohort studies that monitored children’s growth over several months, a progressive increase in the frequency of nutritional deviations identified by different anthropometric indices was observed, indicating a rising prevalence of malnutrition and growth impairment with advancing age. Low length-for-age is defined as a height/length-for-age measurement below − 2 standard deviations from the median of the WHO Child Growth Standards. This condition may result from inadequate nutrition during early childhood, compounded by the exacerbation of common clinical conditions in this population, and may contribute to impairments in cognitive development and growth later in life (Suryawan et al., 2021; World Health Organization, 2006; UNICEF et al., 2023). Stunting prevalence showed considerable magnitude, ranging between 14.3% and 57%. Notably, cohort studies reporting data at multiple time points showed a worsening trend, with stunting prevalence nearly doubling over time: rates rose from 29% to 57% (Tavares et al., 2022) and from 30.9% to 56% (Lopes, 2018) (Table 2).

The anthropometric data presented showed that underweight varied between 14.3% and 54.4% (Table 2). On the other hand, Santos et al. (2019a) described the feeding practices of babies with microcephaly caused by the CZS and found a proportion of 1.5% of children with overweight. Newborns presented underweight in early assessments (Freitas et al., 2020; Oliveira et al., 2020), with higher proportions observed at later follow-up (Aguiar et al., 2022; Lopes, 2018; Santos et al., 2019a). In a survey of 35 newborns with microcephaly, 40% were underweight (Oliveira et al., 2020), while Aguiar et al. (2022) found that 52% of a population aged up to 3 years were underweight. Only one study identified excess weight by the weight-for-age indicator (Santos et al., 2019a).

It is noteworthy that overweight, defined by weight-for-height, was reported in four of the included studies, with proportions ranging from 4.6% (Santos et al., 2019a) to 57% (Coelho et al., 2019). Only seven studies included evaluated BMI-for-age.

**Fig. 2:**
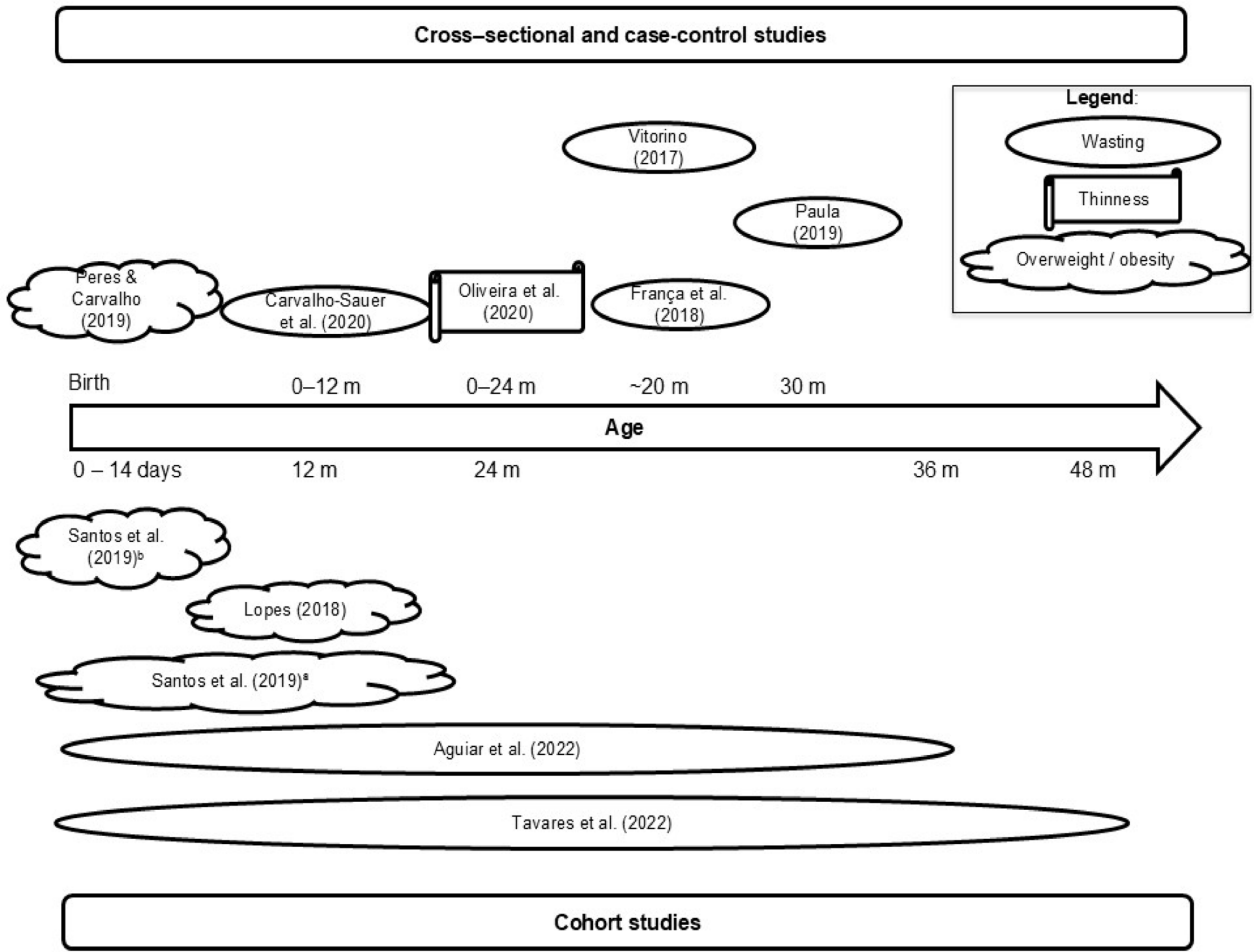
Distribution of included studies according to design, age range evaluated, and main findings

As illustrated in Fig. 2, cross-sectional and case-control studies focused predominantly on the first 30 months of life, whereas cohort studies encompassed a broader follow-up period, ranging from the first 14 days to 48 months of age. At birth and during the first days of life, some children were already classified as overweight or obese; however, over time, a progressive increase in the frequency of wasting was observed with advancing age.

Regarding the BMI-for-age index, two relevant nutritional deviations were identified: underweight and obesity. Among the studies included in this review that reported BMI data, the prevalence of wasting ranged from 14.3% to 48%, while the proportion at risk of overweight varied between 4.8% and 68.6% within the samples. In children aged less than 24 months, 24.6% presented wasting and 6.2% were overweight/obesity (Santos et al., 2019a) (Table 2). The impact of inadequate nutritional status, evidenced by underweight or overweight, and stunting, brings a wide range of subsequent consequences on their development. Malnutrition and overweight/obesity in the first 60 months after birth affect their cognitive neurodevelopment later, compromising growth and learning (Suryawan et al., 2021). The double burden of malnutrition consists of both undernutrition and overweight and obesity.

For the weight-for-height index, studies included in this review found both wasting (4.3% to 37.1%) and overweight (4.6% to 57.2%) (Coelho et al., 2019; Lopes, 2018; Paula, 2019; Santos et al., 2019a) (Table 2). Children in the CZS group presented significantly lower mean weight and height values compared with those without CZS, with differences reaching up to two standard deviations (França et al., 2018). This group also exhibited a progressive reduction in weight-for-height z-scores, an important indicator for assessing acute malnutrition according to the WHO (World Health Organization, 2006). Nevertheless, some included studies reported a higher prevalence of risk of being overweight than of wasting, suggesting the coexistence of contrasting nutritional deviations (Coelho et al., 2019; Lopes, 2018).

As noted, the ranges for malnutrition indices are quite broad (e.g., stunting from 14.3% to 57.1%), which could indicate significant differences between the studies or populations. Some limitations and biases can be highlighted since not all the studies included evaluated or described the process of measuring the weight and height of children. It is necessary to describe how the measurements were obtained or estimated, which may influence the accuracy of the results. Additionally, there is a need for studies with longer follow-up on this population. This variability can make it difficult to pinpoint typical growth deviations.

### Factors Related to Nutritional Deviations and Other Findings

The included studies identified variables potentially associated with the nutritional deviations affecting children with CZS. Among them, dietary practices stand out, such as the duration and presence of exclusive breastfeeding, dietary diversity, the high consumption of processed and ultra-processed foods (UPF), the route of administration of the diet, and clinical complications, such as dysphagia, constipation, acid reflux, and seizures (Table 2).

Carvalho-Sauer et al. (2020) found an inverse association between height-for-age at 12 months and dysphagia (*p* = 0.015), and a direct association between the duration of breastfeeding and the weight-for-age z-score at three (*r* = 0.70; *p* = 0.005) and six months (*r* = 0.54; *p* = 0.025). Newborns of mothers without symptoms of the virus had significantly higher mean z-scores than those born to symptomatic mothers (*p* = 0.036) (Santos et al., 2019b). The presence of microcephaly at birth was associated with birth height (*p* = 0.001), birth weight (*p* = 0.001), and BMI in the second year of life (*p* = 0.04). In this regard, children with microcephaly had a lower z-score for all these associated parameters (Tavares et al., 2022).

#### Feeding Practices

Studies have shown a possible relationship between inappropriate feeding practices and some malnutrition (Aguiar et al., 2022; Lopes, 2018; Santos et al., 2019a). It is known that breastfeeding is a key factor regarding child growth and development, with underweight being a possible consequence of early weaning and inadequate complementary feeding (Coelho et al., 2019; Santos et al., 2021b). In the included studies, the duration of exclusive breastfeeding was shorter than six months, a factor that may be associated with stunting (Carvalho-Sauer et al., 2020; Santos et al., 2019b; Vitorino, 2017).

Difficulties regarding breastfeeding and early weaning of children with microcephaly associated with CZS are mainly related to physiological problems, in addition to lower purchasing power, low educational level, and low maternal age. Furthermore, it is important to emphasize that most children with this condition have dysphagia, causing changes in oral motor coordination, swallowing, and sucking, compromising breastfeeding (Ferreira et al., 2018; Leal et al., 2017; Santos et al., 2019b).

In research on 84 children with microcephaly associated with CZS, 42.9% of them did not receive exclusive breastfeeding at any time in their lives. For those who received it, the average duration was 3.3 months. The mean duration of non-exclusive breastfeeding was 5.4 months. Additionally, the introduction of complementary feeding was delayed, occurring at a mean age of 7.1 months, and 57.1% of the sample exhibited some degree of feeding difficulty (Lopes, 2018). From this perspective, Santos et al. (2019a) identified that approximately 80% of 65 children with CZS did not receive exclusive breastfeeding until the sixth month after birth. Furthermore, 52.3% of children aged 12–23 months were already consuming UPF.

Ingesting UPF and high-energy-density foods in childhood may be related to the nutritional deviations discussed in this review, as well as a worse prognosis The excessive consumption of UPF has negative effects on several areas of child health, including nutritional indicators such as excess weight, adiposity measures, inappropriate feeding practices, and diets with low nutritional quality, in addition to metabolic alterations such as increased glycemic levels and lipid profile. Additionally, it can increase the risk of illnesses such as depression, attention deficit hyperactivity disorder, dental caries, respiratory diseases, and toxicity, with a high urinary concentration of toxic plastic compounds. These adverse child health outcomes underscore the need to reduce the consumption of UPF (Oliveira et al., 2022).

The relationship between growth and the feeding route was also evidenced. Children with neurological problems have significant challenges regarding feeding, and the severity of these difficulties is directly related to the level of neurological impairment. This is because adequate food intake depends on sensory, cognitive, and psychosocial experiences, which may be impaired in these patients. In addition, these children may have gastrointestinal dysmotility and be unable to communicate hunger or seek food between main meals. Due to these factors, alternative feeding methods may be necessary to ensure adequate nutrition, including temporary measures for children (cup, spoon, syringe, or lactation aid) and long-term medical interventions (nasogastric tubes, jejunostomy, and parenteral nutrition) (Paula, 2019; Penagini et al., 2015).

One study included in this review showed that children fed orally had approximately twice the prevalence of underweight and wasting compared with those receiving alternative feeding methods. In contrast, excess weight-for-height was three times more frequent among children using alternative feeding routes. A moderate and statistically significant correlation was also observed between the duration of alternative feeding and anthropometric indices, indicating that longer use was associated with higher weight-for-age (*r* = 0.433; *p* < 0.05) and weight-for-height (*r* = 0.544; *p* < 0.01) z-scores (Paula, 2019). These findings suggest that nutritional status may be influenced by the feeding route; however, the complexity of each child’s nutritional and clinical needs must be carefully considered (Goyal et al., 2019).

#### Clinical Intercurrences

Dysphagia was a condition widely found and with significant values in almost all references included in this review, appearing as a feeding difficulty of children that may be related to underweight and stunting (Leal et al., 2017). Severe dysphagia is commonly found in children with microcephaly, especially from the third month of life onwards (Oliveira et al., 2024; Sociedade Brasileira de Motilidade Digestiva e Neurogastroenterologia, n.d.).

Studies show the presence of dysphagia between 41.2% and 60% in babies aged 0 to 24 months, associated with the height/age index at 12 months (*p* = 0.0148), and in preschoolers with CZS, which also showed high diet intolerance, gastric dysmotility, and nutritional deviations (Almeida et al., 2021; Oliveira et al., 2020; Silva et al., 2025).

Another finding was constipation, which was diagnosed in 41.7% of children with CZS up to 18 months of age (Vitorino, 2017). Constipation is one of the functional diseases of the intestine, becoming a complication in children with neurological deficits, resulting in slower peristalsis and, consequently, dry fecal matter, which is compounded by inadequate water intake, a consequence of dysphagia (Faleiros-Castro & Paula, 2013; Oliveira et al., 2020; Sadeghvand et al., 2025). In a study of preschoolers, 55.5% had constipation, and their fluid intake was inadequate, averaging 0.7 L per day, even though the recommendation is 1.3 to 1.7 L per day (Moura da Silva et al., 2016; Mello et al., 2010).

It was found that 23% of the children examined had gastroesophageal reflux disease. Seizures were reported in 53.5% of the analyzed children aged up to 12 months and in 69.0% of those aged up to 18 months (Oliveira et al., 2020; Santos et al., 2019a; Vitorino, 2017).

## Conclusions

Despite the methodological limitations of the studies analyzed, problems in association measurements, and possible heterogeneity caused by the combination of data from studies with different designs and methodologies, making it challenging to reach definitive conclusions, it was possible to perceive that the double burden of malnutrition is present in children affected by CZS, and dietary practices may be related to these events. Therefore, it is necessary to conduct cohort studies, with adequate follow-up and appropriate association measures, to verify how breast milk and the child’s clinical conditions can interfere with or prevent the occurrence of malnutrition in childhood.

The systematization of a nutritional care and assistance protocol in clinical practice, as well as in professional training, may enhance nutritional surveillance and support the implementation of effective actions aimed at improving the nutritional status and quality of life of children with CZS.
